# Timing of angiography and outcomes in patients with non-ST-segment elevation myocardial infarction: Insights from the evaluation and management of patients with acute chest pain in China registry

**DOI:** 10.3389/fcvm.2022.1000554

**Published:** 2022-10-20

**Authors:** Yu Han, Shukun Sun, Bao Qiao, Han Liu, Chuanxin Zhang, Bailu Wang, Shujian Wei, Yuguo Chen

**Affiliations:** ^1^Department of Emergency and Chest Pain Center, Qilu Hospital of Shandong University, Jinan, China; ^2^Clinical Research Center for Emergency and Critical Care Medicine of Shandong Province, Qilu Hospital of Shandong University, Jinan, China; ^3^Key Laboratory of Emergency and Critical Care Medicine of Shandong Province, Qilu Hospital of Shandong University, Jinan, China; ^4^Key Laboratory of Cardiopulmonary-Cerebral Resuscitation Research of Shandong Province, Qilu Hospital of Shandong University, Jinan, China; ^5^Clinical Trial Center, Qilu Hospital of Shandong University, Jinan, China

**Keywords:** non-ST-segment-elevation myocardial infarction, coronary angiography, percutaneous coronary intervention, major adverse cardiac events, mortality

## Abstract

**Objective:**

Although an invasive strategy has been recommended within 24 h for patients with non-ST-segment elevation myocardial infarction (NSTEMI), the optimal timing of the invasive strategy remains controversial. We sought to investigate the association between the different timings of invasive strategies and clinical outcomes in patients with NSTEMI.

**Materials and methods:**

Patients admitted with NSTEMI from the Evaluation and Management of Patients with Acute ChesT pain in China (EMPACT) registry between January 2016 and September 2017 were included. The primary outcomes were major adverse cardiac events (MACEs) within 30 days. Multivariable logistic regression was performed to assess independent risk factors for MACEs.

**Results:**

A total of 969 patients with NSTEMI from the EMPACT Registry were eligible for this study. Coronary angiography (CAG) was performed in 501 patients [<24 h, *n* = 150 (15.5%); ≥ 24 h, *n* = 351 (36.2%)]. The rate of MACEs at 30 days in all patients was 9.2%, including 54 (5.6%) deaths. Patients who underwent CAG had a lower rate of MACEs and mortality than those who did not receive CAG (MACEs: 5.6% vs. 13.0%, *P* < 0.001; mortality: 1.6% vs. 9.8%, *P* < 0.001). Nonetheless, no statistically significant difference was found in the rates of MACEs and mortality between the early (< 24 h) and delayed (≥ 24 h) CAG groups. Older age (OR: 1.036, 95% CI: 1.007, 1.065, *P* = 0.014), and acute heart failure (OR: 2.431, 95% CI: 1.244, 4.749, *P* = 0.009) increased the risk of MACEs and protective factors were underwent CAG (OR: 0.427, 95% CI: 0.219, 0.832, *P* = 0.012) or PCI (OR: 0.376, 95% CI: 0.163, 0.868, *P* = 0.022). In the multilevel logistic regression, older age (OR: 0.944, 95% CI: 0.932, 0.957, *P* < 0.001), cardiogenic shock (OR: 0.233, 95% CI: 0.079, 0.629, *P* = 0.009), pulmonary moist rales (OR: 0.368, 95% CI: 0.197, 0.686, *P* = 0.002), and prior chronic kidney disease (OR: 0.070, 95% CI: 0.018, 0.273, *P* < 0.001) was negatively associated with CAG.

**Conclusion:**

This real-world cohort study of NSTEMI patients confirmed that the early invasive strategy did not reduce the incidence of MACEs and mortality within 30 days compared with the delayed invasive strategy in NSTEMI patients.

## Introduction

Acute myocardial infarction (AMI) has a significant worldwide health impact and is a leading cause of mortality and disability (1–3). Based on electrocardiogram (ECG) characteristics, AMI can be classified into ST-segment elevation myocardial infarction (STEMI) and non-ST-segment elevation myocardial infarction (NSTEMI). The risk of recurrent cardiovascular events is higher in both STEMI and NSTEMI patients, but NSTEMI patients have higher long-term mortality and greater cardiovascular risk than STEMI patients (4, 5). Furthermore, the proportion of patients with NSTEMI has increased since the 1990s and NSTEMI has become the leading cause of emergency admissions for AMI patients in Europe, the USA, and China (6–10). NSTEMI patients are older and more often female than STEMI patients (11). In addition, the clinical manifestations of NSTEMI patients are diverse, ranging from asymptomatic patients to those with persistent myocardial ischemia, heart failure (HF), cardiogenic shock, and even cardiac arrest. Therefore, the management of NSTEMI is complicated. Recently, an early invasive strategy (within the first 24 h after hospital admission) was recommended for NSTEMI patients, but the optimal timing of the invasive strategy remains to be further explored (12).

Few studies have focused on the optimal timing of invasive strategies for the Chinese NSTEMI population. In the present study, we sought to investigate the association between the different timings of invasive strategies and clinical outcomes in NSTEMI patients using data from the Evaluation and Management of Patients with Acute ChesT pain in China (EMPACT) registry (13).

## Materials and methods

### Study population

The methods of the EMPACT registry (NCT02536677) have been previously described (13). In short, EMPACT was a multicenter prospective registry collecting the clinical characteristics and outcomes of emergency department (ED) patients experiencing acute chest pain and acute coronary syndrome (ACS)-related symptoms from 22 representative public hospitals in Shandong Province, China. Consecutive NSTEMI cases enrolled in the EMPACT registry from January 1, 2016, to September 30, 2017, were eligible for this study. The diagnosis of NSTEMI has been covered in extensive detail, including European and US clinical practice guidelines (12, 14). In short, cardiac troponin elevation with ischemic symptoms or ECG changes but without new persistent ST-segment elevation are defined as NSTEMI. Elevated troponin is defined as a measurement exceeding the 99th percentile of the upper reference limit. Eventually, 969 patients with NSTEMI were eligible for this study. The Ethics Committee of Qilu Hospital of Shandong University approved the study (No.2015-058), and all patients provided written informed consent.

### Timing of angiography and percutaneous coronary intervention

For the present analysis, patients were categorized into 3 groups according to the time of their first coronary angiography (CAG) after admission: no, early (<24 h), or delayed (≥24 h) CAG. Patients undergoing percutaneous coronary intervention (PCI) were categorized into 2 groups according to the time of their first PCI after admission: the early PCI group (<24 h), and the delayed PCI group (≥24 h).

### Follow-up and definitions of outcomes

Follow-up began at discharge and lasted 30 days for outcome confirmation. The primary outcomes were major adverse cardiac events (MACEs) within 30 days, which is a composite of death from all causes, non-fatal myocardial infarction (MI), urgent revascularization, stroke, cardiac arrest, and cardiogenic shock.

### Statistical analysis

Categorical variables were expressed as numbers (percentages) and compared by the chi-square test or Fisher’s exact test as appropriate. Continuous variables were described as medians (interquartile ranges) and compared by the Mann-Whitney *U*-test.

To evaluate the relationship between the different timings of invasive strategies and the incidence of MACEs, we constructed univariable and multivariate logistic regression modes. Any variables having *P* < 0.1 in the univariate analyses were included in the multivariate regression analysis. Thus, multivariable models included the following variables: undergoing CAG, undergoing PCI, undergoing delayed CAG, undergoing delayed PCI, older age, body mass index (BMI), acute heart failure (HF), systolic blood pressure, cardiogenic shock, diabetes, prior HF, prior stroke, and pulmonary moist rales. Secondary analyses examined the associations between the different timings of invasive strategies and MACEs within prespecified subgroups. Subgroups were selected to focus on the types of patients expected to benefit (or harm) from the early invasive strategy, including age (< 75 years or ≥ 75 years), sex, and the presence of heart failure.

Factors associated with CAG occurrence were assessed by a multiple logistic regression initially including all the variables having *P* < 0.1 in the univariate analyses and then we applied a stepwise backward selection of the variables which remained significant (*P* < 0.05). The following variables were included in the multivariate model: older age, cardiogenic shock, pulmonary moist rales, and prior CKD. Sensitivity analyses were performed to test the stability of results by removing patients with cardiogenic shock, and patients with cardiogenic shock and prior CKD.

Statistical analyses were performed using SAS software, version 9.4 (SAS Institute, Cary, NC, US). Two-sided *P*-values < 0.05 were considered statistically significant.

## Results

### Baseline characteristics

**FIGURE 1:**
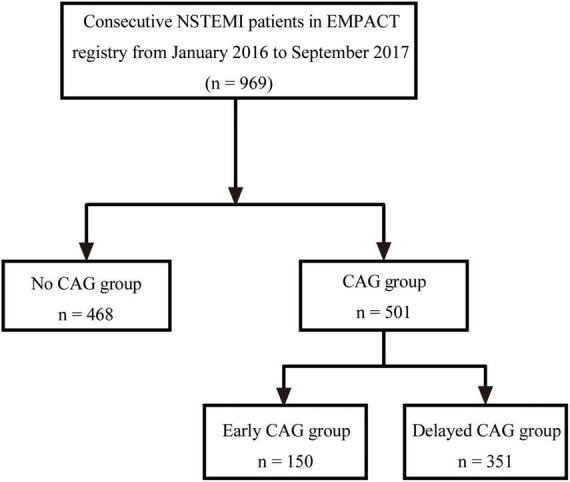
Flow diagram of patient selection. AMI, acute myocardial infarction; STEMI, ST-segment elevation myocardial infarction; UA, unstable angina; NSTEMI, non-ST segment elevation myocardial infarction; CAG, coronary angiography.

A total of 969 NSTEMI patients were eligible from the EMPACT registry according to inclusion and exclusion criteria (Figure 1). 150 NSTEMI patients (15.5%) underwent early CAG and 351 NSTEMI patients (36.2%) received delayed CAG (Supplementary Table 1). 332 NSTEMI patients (34.3%) underwent PCI. Among them, 103 underwent early PCI and 229 underwent delayed PCI (Supplementary Table 2). Table 1 shows the clinical characteristics of NSTEMI patients. 355 (36.6%) patients were females. The mean age was 67 years old, and 28% of patients were current smokers. Comorbidities of diabetes, hypertension, and hyperlipidemia accounted for 25.5, 60.1, and 9.5% of all patients, respectively. On admission, the mean blood pressure was 146/86 mm Hg and the mean heart rate was 79 beats/min. Of all patients, 12.7% experienced acute HF, and 2.3% experienced cardiogenic shock. Patients who did not undergo CAG were more likely to be female (42.5% vs. 31.1%, *P* < 0.001) and older (71.5 years old vs. 64 years old, *P* < 0.001).

**TABLE 1:** Baseline clinical characteristics for NSTEMI patients.

	All (*n* = 969)	NO CAG (*n* = 468)	CAG (*n* = 501)	*P*-value
** Demographic**				
Female sex, *n* (%)	355 (36.6)	199 (42.5)	156 (31.1)	<0.001
Age, mean (*SD*), y	67 (60, 76)	71.5 (63, 80)	64 (55, 71)	<0.001
BMI (*SD*), kg/m^2^	24.7 (22.9, 26.9)	24.3 (22.5, 26.3)	25.1 (23.2, 27.3)	<0.001
Current smoker	271 (28.0)	105 (22.4)	166 (33.1)	<0.001
** Medical history, *n* (%)**				
premature CHD family history	105 (10.8)	38 (8.1)	67 (13.4)	0.009
Prior MI	203 (20.9)	122 (26.1)	81 (16.2)	<0.001
Prior PCI	116 (12.0)	51 (10.9)	65 (13.0)	0.320
Prior CABG	32 (3.3)	22 (4.7)	10 (2.0)	0.019
Diabetes	247 (25.5)	135 (28.8)	112 (22.4)	0.021
Hypertension	582 (60.1)	278 (59.4)	304 (60.7)	0.685
Hyperlipidemia	92 (9.5)	41 (8.8)	51 (10.2)	0.451
Prior HF	35 (3.6)	27 (5.8)	8 (1.6)	0.001
Prior CKD	24 (2.5)	21 (4.5)	3 (0.6)	<0.001
Chronic lung disease	43 (4.4)	31 (6.6)	12 (2.4)	0.001
Peripheral arterial disease	5 (0.5)	5 (1.1)	0 (0)	0.026
Prior stroke	125 (12.9)	79 (16.9)	46 (9.2)	<0.001
** On presentation**				
Systolic blood pressure (mm Hg), mean (*SD*)	146 (126, 165)	143 (125, 163.5)	148 (127, 166)	0.063
Diastolic blood pressure (mm Hg), mean (*SD*)	86 (74, 98.5)	82 (71, 95.5)	88 (76, 100)	<0.001
Heart rate (beats/min), mean (*SD*)	79 (68, 93)	81 (70, 96)	77 (66, 89)	<0.001
Cardiogenic shock, *n* (%)	22 (2.3)	17 (3.6)	5 (1.0)	0.006
HF, *n* (%)	123 (12.7)	69 (14.7)	54 (10.8)	0.064
Abnormal heart auscultation, *n* (%)	101 (10.4)	62 (13.2)	39 (7.8)	0.005
Pulmonary moist rales, *n* (%)	71 (7.3)	56 (12.0)	15 (3.0)	<0.001
Lower extremity edema, *n* (%)	47 (4.9)	32 (6.8)	15 (3.0)	0.005
**Biochemical indices were positive, *n* (%)**				
D-dimer	125 (33.4)	85 (49.7)	40 (19.7)	<0.001
BNP	112 (45.0)	59 (49.6)	53 (40.8)	0.163

NSTEMI, non-ST segment elevation myocardial infarction; CAG, coronary angiography; BMI, body mass index; CHD, coronary heart disease; MI, myocardial infarction; PCI, percutaneous coronary intervention; CABG, coronary artery bypass grafting; HF, heart failure; CKD, chronic kidney disease; BNP, brain sodium peptide.

### Emergency medication

Aspirin, adenosine diphosphate (ADP) receptor antagonists, and Low molecular weight heparins were given in 49.7, 41.5, and 27.2% of all NSTEMI patients, respectively. NSTEMI patients who did not receive CAG were less likely to receive aspirin (46.8% vs. 52.5%, *P* = 0.076), ADP receptor antagonist (35.9% vs. 46.7%, *P* = 0.001), and low molecular weight heparin (22.9% vs. 31.3%, *P* = 0.003) than NSTEMI patients who received CAG. NSTEMI patients who underwent CAG were more likely to receive statins (34.1% vs. 23.9%, *P* < 0.001), nitrate esters (51.1% vs. 48.7%, *P* = 0.459), and Chinese patent drugs (38.5% vs. 31.8%, *P* = 0.030) than NSTEMI patients who did not undergo CAG (Table 2).

**TABLE 2:** Emergency medication for NSTEMI patients in the EDs.

	ALL (*n* = 969)	NO CAG (*n* = 468)	CAG				*P*-value (yes vs. no)
			Total (*n* = 501)	Early CAG (*n* = 150)	Delayed CAG (*n* = 351)	*P-*value (Early vs. delayed)	
Aspirin, *n* %	482 (49.7)	219 (46.8)	263 (52.5)	77 (51.3)	186 (53.0)	0.734	0.076
ADP receptor Antagonists, *n* %	402 (41.5)	168 (35.9)	234 (46.7)	72 (48)	162 (46.2)	0.704	0.001
Statins, *n* %	283 (29.2)	112 (23.9)	171 (34.1)	46 (30.7)	125 (35.6)	0.285	<0.001
Nitrate esters, *n* %	484 (49.9)	228 (48.7)	256 (51.1)	69 (46.0)	187 (53.3)	0.136	0.459
LMWH, *n* %	264 (27.2)	107 (22.9)	157 (31.3)	19 (12.7)	138 (39.3)	<0.001	0.003
Chinese patent drug, *n* %	342 (35.3)	149 (31.8)	193 (38.5)	34 (22.7)	159 (45.3)	<0.001	0.030

NSTEMI, non-ST segment elevation myocardial infarction; EDs, emergency departments; CAG, coronary angiography; ADP, adenosine diphosphate; LMWH, low molecular weight heparin.

### Thirty-days outcomes

The rate of MACEs in all patients was 9.2%, including 54 (5.6%) deaths (Table 3). Patients who underwent CAG had a lower rate of MACEs and mortality than those who did not receive CAG (MACEs: 5.6% vs. 13%, *P* < 0.001; mortality: 1.6% vs. 9.8%, *P* < 0.001). However, there was no statistically significant difference in the rates of MACEs and mortality between the early and delayed CAG groups (MACEs: 6.7% vs. 5.1%, *P* = 0.492; mortality: 2.0% vs. 1.4%, *P* = 0.701). Moreover, when 30-day outcomes were compared among patients who received PCI at different times, there were no statistically significant differences either (Supplementary Table 3). There were also no statistically significant differences in the rates of MACEs of NSTEMI patients undergoing early CAG vs. delayed CAG when subgroup analysis was performed according to age, sex, or the presence of HF (Supplementary Table 4).

**TABLE 3:** 30 days outcomes of patients with NSTEMI undergoing CAG.

	ALL (*n* = 969)	NO CAG (*n* = 468)	CAG				*P*-value (yes vs. no)
			Total (*n* = 501)	Early CAG (*n* = 150)	Delayed CAG (*n* = 351)	*P*-value (Early vs. delayed)	
All, *n* %	89 (9.2)	61 (13.0)	28 (5.6)	10 (6.7)	18 (5.1)	0.492	<0.001
Death, *n* %	54 (5.6)	46 (9.8)	8 (1.6)	3 (2)	5 (1.4)	0.701	<0.001
Myocardial infarction, *n* %	9 (0.9)	3 (0.6)	6 (1.2)	1 (0.7)	5 (1.4)	0.674	0.508
Emergency revascularization, *n* %	2 (0.2)	0 (0)	2 (0.4)	1 (0.7)	1 (0.3)	0.510	0.500
Cardiogenic shock, *n* %	33 (3.4)	21 (4.5)	12 (2.4)	5 (3.3)	7 (2.0)	0.356	0.073
Cardiac arrest/ventricular Fibrillation, *n* %	36 (3.7)	28 (6)	8 (1.6)	5 (3.3)	3 (0.9)	0.056	<0.001
Stroke, *n* %	10 (1.0)	4 (0.9)	6 (1.2)	2 (1.3)	4 (1.1)	1.000	0.754

NSTEMI, Non-ST segment elevation myocardial infarction; CAG, coronary angiography.

### Bleeding and procedural complications

Bleeding complications were shown in Supplementary Table 5. There was no statistically significant difference in the rates of bleeding complications between the early and delayed CAG groups (11.3% vs. 6.8%, *P* = 0.109). Moreover, no statistically significant difference was found in the rates of procedural complications between the early and delayed PCI groups (14.6% vs. 10.5%, *P* = 0.357) (Supplementary Table 6).

### Independent predictors of the rate of major adverse cardiac events in patients with non-ST-segment elevation myocardial infarction

**FIGURE 2:**
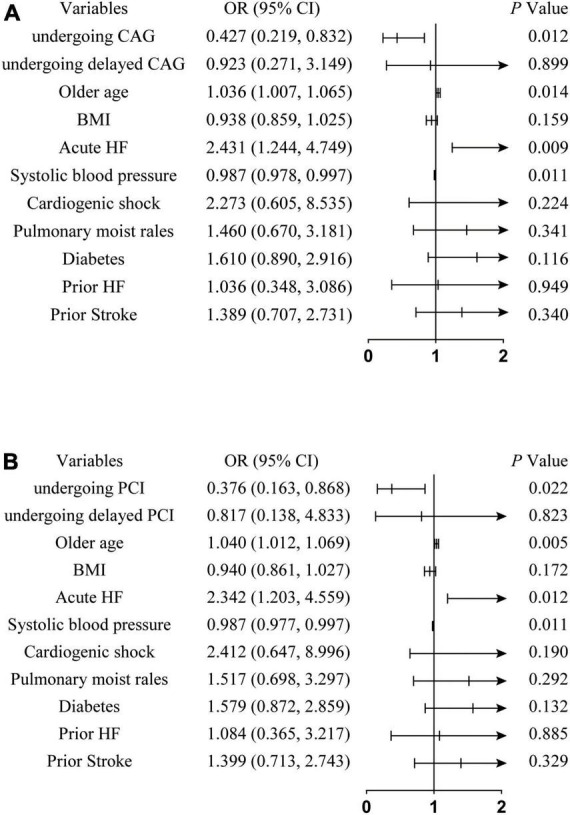
Forest plot of odds ratio for the rate of MACEs of NSTEMI patients. **(A)** Represents the independent effects of undergoing CAG on the incidence of MACEs; **(B)** represents the independent effects of undergoing PCI on the incidence of MACEs. NSTEMI, non-ST segment elevation myocardial infarction; MACEs, major adverse cardiac events; OR, odds ratio; CI, conference interval; CAG, coronary angiography; HF, heart failure; BMI, body mass index; PCI, percutaneous coronary intervention.

Table 4 and Figure 2 show the logistic regression model with the odds ratio (OR) and 95% confidence interval (CI) of the predictors of the rate of MACEs. Older age (OR: 1.036, 95% CI: 1.007, 1.065, *P* = 0.014) and acute HF (OR: 2.431, 95% CI: 1.244, 4.749, *P* = 0.009) increased the risk of MACEs and protective factors were associated with CAG (OR: 0.427, 95% CI: 0.219, 0.832, *P* = 0.012) or PCI (OR: 0.376, 95% CI: 0.163, 0.868, *P* = 0.022). The timing of undergoing CAG (OR: 0.923, 95% CI: 0.271, 3.149, *P* = 0.899) or PCI (OR: 0.817, 95% CI: 0.138, 4.833, *P* = 0.823), BMI (OR: 0.938, 95% CI: 0.859, 1.025, *P* = 0.159), cardiogenic shock (OR: 2.273, 95% CI: 0.605, 8.535, *P* = 0.224), diabetes (OR: 1.610, 95% CI: 0.890, 2.916, *P* = 0.116), prior HF (OR: 1.036, 95% CI: 0.348, 3.086, *P* = 0.949), prior stroke (OR: 1.389, 95% CI: 0.707, 2.731, *P* = 0.304), and pulmonary moist rales (OR: 1.460, 95% CI: 0.670, 3.181, *P* = 0.341) are not independent influences on the rate of MACEs.

**TABLE 4:** Univariate analysis of the rate of MACEs of NSTEMI patients.

Variables	OR	95% CI	*P-*value
CAG	0.246	0.134, 0.452	<0.001
Age	1.062	1.035, 1.088	<0.001
Sex	1.518	0.908, 2.536	0.112
Current smoker	0.588	0.308, 1.120	0.106
BMI	0.898	0.827, 0.976	0.012
Heart rate	1.001	0.990, 1.012	0.823
Systolic blood pressure	0.985	0.976, 0.993	<0.001
Diastolic blood pressure	0.976	0.963, 0.990	0.001
Cardiogenic shock	7.425	2.910, 18.95	<0.001
Abnormal heart auscultation	1.272	0.588, 2.752	0.542
Acute HF	3.626	2.053, 6.404	<0.001
Pulmonary moist rales	3.378	1.708, 6.681	<0.001
Lower extremity edema	0.980	0.296, 3.247	0.973
Prior CABG	1.512	0.448, 5.107	0.506
Prior CKD	2.108	0.611, 7.265	0.238
Chronic lung disease	0.332	0.045, 2.451	0.280
Diabetes	1.885	1.109, 3.204	0.019
Prior HF	2.517	0.942, 6.730	0.066
premature CHD family history	0.695	0.272, 1.773	0.446
Prior MI	1.559	0.882, 2.757	0.127
Prior stroke	2.490	1.364, 4.544	0.003

NSTEMI, non-ST segment elevation myocardial infarction; MACEs, major adverse cardiac events; OR, odds ratio; CI, confidence interval; CAG, coronary angiography; BMI, body mass index; HF, heart failure; CABG, coronary artery bypass grafting; CKD, chronic kidney disease; MI, myocardial infarction.

### Independent predictors of undergoing coronary angiography in patients with non-ST-segment elevation myocardial infarction

**FIGURE 3:**
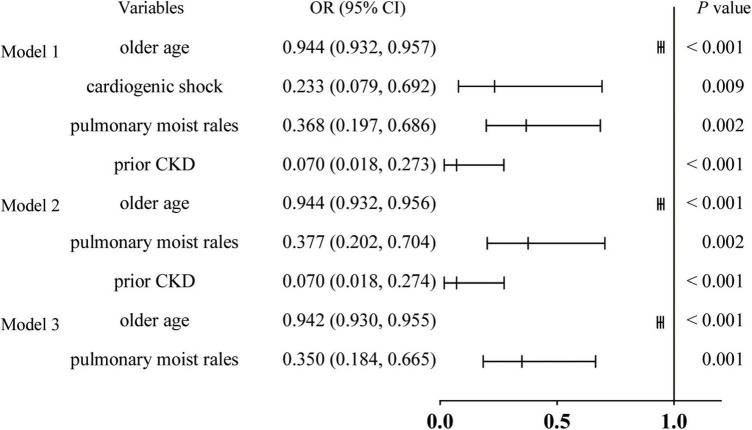
Forest plot of odds ratio for risk factors of undergoing CAG. Model 1, all patients; Model 2, remove patients with cardiogenic shock; and Model 3, remove patients with cardiogenic shock and CKD; NSTEMI, non-ST segment elevation myocardial infarction; CAG, coronary angiography; OR, odds ratio; CI, conference interval; CKD, chronic kidney disease.

Table 5 and Figure 3 show the logistic regression model with OR (95% CI) of the predictors of undergoing CAG. Older age (OR: 0.944, 95% CI: 0.932, 0.957, *P* < 0.001), cardiogenic shock (OR: 0.233, 95% CI: 0.079, 0.629, *P* = 0.009), pulmonary moist rales (OR: 0.368, 95% CI: 0.197, 0.686, *P* = 0.002), and prior CKD (OR: 0.070, 95% CI: 0.018, 0.273, *P* < 0.001) were negatively associated with CAG. The OR value of the variables in the three models did not change significantly, confirming the stability of the logistic regression model.

**TABLE 5:** Univariate analysis of whether NSTEMI patients undergo CAG.

Variables	OR	95% CI	*P-*value
Age	0.944	0.933, 0.956	<0.001
Sex	0.611	0.470, 0.795	<0.001
Current smoker	1.713	1.287, 2.280	<0.001
BMI	1.078	1.036, 1.123	<0.001
Heart rate	0.987	0.982, 0.993	<0.001
Systolic blood pressure	1.004	0.999, 1.008	0.099
Diastolic blood pressure	1.012	1.005, 1.020	0.001
Cardiogenic shock	0.267	0.098, 0.731	0.010
Abnormal heart auscultation	0.553	0.362, 0.843	0.006
Acute HF	0.699	0.477, 1.022	0.065
Pulmonary moist rales	0.227	0.127, 0.407	<0.001
Lower extremity edema	0.421	0.225, 0.787	0.007
Prior CABG	0.413	0.193, 0.882	0.022
Prior CKD	0.128	0.038, 0.433	0.001
Chronic lung disease	0.346	0.176, 0.682	0.002
Diabetes	0.710	0.531, 0.949	0.021
Prior HF	0.265	0.119, 0.589	0.001
premature CHD family history	1.747	1.148, 2.658	0.009
Prior MI	0.547	0.399, 0.749	<0.001
Prior stroke	0.498	0.338, 0.734	<0.001

NSTEMI, non-ST segment elevation myocardial infarction; CAG, coronary angiography; OR, odds ratio; CI, confidence interval; BMI, body mass index; HF, heart failure; CABG, coronary artery bypass grafting; CKD, chronic kidney disease; MI, myocardial infarction.

## Discussion

Based on the EMPACT registry, the present study revealed that undergoing CAG or PCI was beneficial in improving the short-term prognosis (30 days) of patients with NSTEMI, while the timing of undergoing CAG or PCI was not associated with the rate of MACEs.

Several randomized, controlled trials (RCTs) have addressed the optimal timing of invasive strategies for NSTEMI patients. Both TIMACS (Timing of Intervention in Acute Coronary Syndromes) and VERDICT (Very Early vs. Deferred Invasive Evaluation Using Computerized Tomography) trials confirmed that an early invasive strategy improved clinical outcomes in non-ST-segment elevation acute coronary syndrome (NSTE-ACS) patients with a GRACE score > 140 (15, 16). Based on the above evidence, the European society of cardiology (ESC) guideline recommends invasive treatment within 24 h for NSTEMI patients (12). The present study did not specifically focus on the GRACE risk score of NSTEMI patients, but it assessed the association between the timing of invasive strategies and clinical outcomes in unselected NSTEMI cohorts. The results showed that an early invasive strategy did not reduce the incidence of MACEs or mortality within 30 days compared with delayed invasive strategies. A meta-analysis including 10 trials from 2003 to 2016 has compared early (0.5–14 h) and delayed (18.3–86 h) strategies in 6,397 NSTEMI patients with moderate or high risk (17). Similarly, there was no difference in terms of mortality (4.0% vs. 4.7%; OR: 0.85; 95% CI: 0.67–1.09; *P* = 0.20) or MI (6.7% vs. 7.7%; OR: 0.88; 95% CI: 0.53–1.45; *P* = 0.62) (17). However, another meta-analysis including 14 RCTs (9,637 patients) showed that the early invasive strategy was associated with a lower incidence of MACEs than the delayed invasive strategy (RR: 0.65; 95% CI: 0.49, 0.87; *P* = 0.003) (18). Contradictory results were obtained from our and previous studies. The timing of intervention, outcome indicators, and follow-up time varied between studies. The diagnosis and prognosis of NSTEMI have been improved considerably in recent years with the introduction of high-sensitivity troponin, the use of second-generation drug-eluting stents, and advancements in P2Y12 inhibitors (19–21). The results of some trials were based on previous generation stents and antithrombotic therapy, rendering comparisons between studies difficult. Therefore, it is necessary to further explore the optimal timing of invasive treatment for patients with NSTEMI by designing more refined randomized controlled trials in the future.

The OPERA registry reported that HF and age were predictors of 1-year mortality in NSTEMI patients (22). Similarly, the present study showed that older age and acute HF increased the risk of 30-day MACEs. Park et al. reported that diabetes, major bleeding, multivessel disease, post-TIMI flow, Killip class, and left ventricular dysfunction were independently associated with the risk of cardiac mortality (within 30 days) in NSTEMI patients (23). In our study, however, diabetes was not an independent influence on the incidence of MACEs. We did not assess multivessel disease, post-PCI TIMI flow, Killip class, and left ventricular dysfunction in our analysis because related information was not available in the registry. These variables may have an impact on clinical outcomes. Thus, well-designed investigations with other variables should be conducted to corroborate the findings of this study.

In this study, only 51.7% of NSTEMI patients underwent CAG, which is lower than that in France (95.0%), Germany (60.2%), and the US (58.0%) (11, 24, 25). This rate was even lower than the rate of revascularization (58.2%) reported in the Improving CCC Project in Chinese NSTE-ACS patients (26). Previous studies have indicated that the invasive treatment of NSTEMI patients is far from standardized in China (26, 27). Therefore, it is necessary to explore the causes to provide opportunities for improvement. Further analysis demonstrated that older age, cardiogenic shock, pulmonary moist rales, and prior CKD were associated with CAG. The treatment strategy for older patients with NSTEMI can be challenging for clinicians since they are more likely to have atypical symptoms than younger patients (28). A meta-analysis including 3 RCTs with 5-year outcomes showed that patients older than 75 years old benefited from the invasive strategy, while data for patients older than 80 years old were not available (29). Another RCT determined that the invasive strategy was superior to the conservative strategy in reducing combined events in NSTEMI patients older than 80 years old. Moreover, there was no difference between the two strategies in terms of bleeding complications (30). Thus, early invasive treatment represents a safe strategy for the majority of elderly NSTEMI patients.

Current guidelines recommend that NSTEMI patients with CKD undergo appropriate invasive treatment, except for those with advanced CKD (12, 14). However, a smaller proportion of CKD patients underwent invasive treatment, due to previous studies that have shown that NSTEMI patients with CKD or a low glomerular filtration rate were at high risk for surgical complications such as bleeding events, acute kidney injury, and death (31, 32). A study including 12,821 (mean age 86 years old) NSTEMI patients demonstrated that patients undergoing PCI had a significantly lower risk of death than those treated conservatively during the follow-up period (3.2 years), and this finding held in all stages of CKD (33). Therefore, CKD should not be a reason to avoid revascularization for NSTEMI patients.

NSTEMI patients with HF are less likely to receive CAG or PCI than non-HF patients and they have a higher risk of death at 30 days (34). Steg et al. demonstrated a reduction in post-discharge mortality in NSTEMI patients with HF who received invasive therapy, indicating the possibility of widespread use of invasive treatment in this high-risk population (35). Cardiogenic shock is a life-threatening complication in NSTEMI patients (36). In this study, 2.3% of the patients presented with cardiogenic shock. Regardless of the ECG presentation, guidelines recommend early invasive treatment in hemodynamically unstable patients (12, 14). A report from the SHOCK trial showed that approximately two-thirds of NSTEMI patients with cardiogenic shock had triple-vessel lesions (37). Omer et al. explored the clinical outcomes of multivessel vs. culprit vessel-only PCI in NSTEMI patients with multivessel disease and cardiogenic shock (38). The findings indicated that multivessel PCI reduced all-cause in-hospital mortality while leading to more procedural complications (38). The risks associated with perioperative complications could outweigh the benefits of the invasive treatment under some conditions. Therefore, clinicians must consider the patient’s comorbidities, life expectancy, and bleeding risk and consequently recognize those patients who might not benefit from the early invasive treatment.

### Limitations

Several limitations of the present study are as follows. First, as a retrospective study, it only revealed important correlations and could not prove causality. Second, the present study only provided real-world data for the outcomes of NSTEMI patients who underwent invasive treatment at different times. Further studies integrating propensity matching or risk adjustment and randomized prospective controlled studies are necessary to assess whether the prognosis of NSTEMI patients is better with an early invasive strategy than with a delayed invasive strategy. Third, although we performed multivariate logistic regression analysis to overcome the limitations of this retrospective study, the results were still affected by unobserved confounding factors, such as variables that were not included in the registry.

## Conclusion

This real-world cohort study of NSTEMI patients supported that an early invasive strategy did not reduce the incidence of MACEs or mortality within 30 days compared with the delayed invasive strategy in NSTEMI patients.

## Data availability statement

The original contributions presented in this study are included in the article/Supplementary material, further inquiries can be directed to the corresponding author/s.

## Ethics statement

The studies involving human participants were reviewed and approved by the Ethics Committee of Qilu Hospital of Shandong University. The patients/participants provided their written informed consent to participate in this study.

## Author contributions

YH and SW made substantial contributions to the conception of the study. YH, SS, and SW contributed to the design of the work. YH, HL, BW, and SS made substantial contributions to the acquisition and analysis of the data and the interpretation of data. BQ, CZ, and SW drafted the work. SW, YC, and YH revised and edited the draft. All authors approved the submitted version and have agreed to be personally accountable for the author’s contributions and to ensure that questions related to the accuracy or integrity of any part of the work, even ones in which the author was not personally involved, were appropriately investigated, resolved, and the resolution documented in the literature, read, and approved the final manuscript.
