# Supplementary material for: Timing of angiography and outcomes in patients with non-ST-segment elevation myocardial infarction: Insights from the evaluation and management of patients with acute chest pain in China registry
Source: Front Cardiovasc Med. 2022 Oct 20;9:1000554. doi: 10.3389/fcvm.2022.1000554 (PMC9630349; doi:10.3389/fcvm.2022.1000554)
Supplement: Supplementary file 1 [file Data_Sheet_1.pdf]

Supplemental Materials

Supplementary Table 1. Baseline clinical characteristics for patients with NSTEMI undergoing CAG

	Early CAG (n=150)	Delayed CAG (n=351)	*P* Value
Demographic			
Female sex, n (%)	35 (23.3)	121 (34.5)	0.014
Age, mean (SD), y	64 (55, 69)	64 (56, 71)	0.470
BMI, mean (SD), kg/m^2	24.5 (22.6, 26.8)	25.4 (23.4, 27.4)	0.006
Current smoker	61 (40.7)	105 (29.9)	0.019
Medical history, n (%)			
premature CHD family history	17 (11.3)	50 (14.2)	0.381
Prior MI	17 (11.3)	64 (18.2)	0.055
Prior PCI	16 (10.7)	49 (14.0)	0.315
Prior CABG	0 (0)	10 (2.8)	0.037
Diabetes	22 (14.7)	90 (25.6)	0.007
Hypertension	84 (56.0)	220 (62.7)	0.161
Hyperlipidemia	16 (10.7)	35 (10.0)	0.814
Prior HF	3 (2.0)	5 (1.4)	0.701
Prior CKD	0 (0)	3 (0.9)	0.558
Chronic lung disease	4 (2.7)	8 (2.3)	0.758
Peripheral arterial disease	0 (0)	0 (0)	NA
Prior Stroke	12 (8.0)	34 (9.7)	0.549
On presentation			
Systolic blood pressure (mm Hg), mean (SD)	141 (122, 165)	150 (130, 167)	0.083
Diastolic blood pressure (mm Hg), mean (SD)	88 (73, 100)	88 (78, 100)	0.320
Heart rate (beats/min), mean (SD)	73 (62, 85)	78 (68, 90)	0.003
Cardiogenic shock, n (%)	2 (1.3)	3 (0.9)	0.638
HF, n (%)	11 (7.3)	43 (12.3)	0.104
Abnormal heart auscultation, n (%)	16 (10.7)	23 (6.6)	0.115
pulmonary moist rales, n (%)	4 (2.7)	11 (3.1)	1.000
lower extremity edema, n (%)	3 (2.0)	12 (3.4)	0.569
Biochemical indices were positive, n (%)			
D-dimer	4 (8.5)	36 (23.1)	0.028
BNP	11 (31.4)	42 (44.2)	0.188

NSTEMI: non-ST segment elevation myocardial infarction; CAG: coronary angiography; PCI: percutaneous coronary intervention; BMI: body mass index; CHD: coronary heart disease; MI: myocardial infarction; CABG: coronary artery bypass grafting; HF: heart failure; CKD: chronic kidney disease; BNP: brain sodium peptide.

Supplementary Table 2. Baseline clinical characteristics for patients with NSTEMI undergoing PCI

	All (n=332)	Early PCI (n=103)	Delayed PCI (n=229)	*P* Value
Demographic				
Female sex, n (%)	99 (29.8)	18 (17.5)	81 (35.4)	0.001
Age, mean (SD), y	63 (56, 70)	63 (54, 68)	63 (57, 71)	0.271
BMI, mean (SD), kg/m^2	25.3 (23.4, 27.3)	24.7 (22.9, 26.9)	25.4 (23.9, 27.5)	0.018
Current smoker	110 (33.1)	43 (41.7)	67 (29.3)	0.025
Medical history, n (%)				
Premature CHD family history	44 (13.3)	12 (11.7)	32 (14)	0.564
Prior MI	49 (14.8)	14 (13.6)	35 (15.3)	0.688
Prior PCI	45 (13.6)	13 (12.6)	32 (14)	0.739
Prior CABG	6 (1.8)	0 (0)	6 (2.6)	0.183
Diabetes	66 (19.9)	13 (12.6)	53 (23.1)	0.026
Hypertension	199 (59.9)	57 (55.3)	142 (62)	0.251
Hyperlipidemia	36 (10.8)	11 (10.7)	25 (10.9)	0.949
Prior HF	5 (1.5)	2 (1.9)	3 (1.3)	0.647
Prior CKD	1 (0.3)	0 (0)	1 (0.4)	1.000
Chronic lung disease	7 (2.1)	2 (1.9)	5 (2.2)	1.000
Peripheral arterial disease	0 (0)	0 (0)	0 (0)	NA
Prior Stroke	27 (8.1)	7 (6.8)	20 (8.7)	0.550
On presentation				
Systolic blood pressure (mm Hg), mean (SD)	148 (127, 167)	142 (122, 162)	150 (132, 170)	0.031
Diastolic blood pressure (mm Hg), mean (SD)	89 (76.5, 100)	87 (73, 100)	90 (79, 101)	0.177
Heart rate (beats/min), mean (SD)	76 (64.5, 88)	70 (59, 81)	78 (68, 89)	< 0.001
Cardiogenic shock, n (%)	3 (0.9)	1 (1)	2 (0.9)	1.000
HF, n (%)	32 (9.6)	7 (6.8)	25 (10.9)	0.239
Abnormal heart auscultation, n (%)	27 (8.1)	14 (13.6)	13 (5.7)	0.015
pulmonary moist rales, n (%)	8 (2.4)	3 (2.9)	5 (2.2)	0.707
lower extremity edema, n (%)	9 (2.7)	2 (1.9)	7 (3.1)	0.726
Biochemical indices were positive, n (%)				
D-dimer	27 (20.8)	2 (6.3)	25 (25.5)	0.020
BNP	32 (42.1)	7 (33.3)	25 (45.5)	0.339

NSTEMI: non-ST segment elevation myocardial infarction; PCI: percutaneous coronary intervention; BMI: body mass index; CHD: coronary heart disease; MI: myocardial infarction; CABG: coronary artery bypass grafting; HF: heart failure; CKD: chronic kidney disease; BNP: brain sodium peptide.

Supplementary Table 3. 30 days outcomes of NSTEMI patients undergoing PCI

	All (n=332)	Early PCI (n=103)	Delayed PCI (n=229)	*P* Value
All, n%	11 (3.3)	4 (3.9)	7 (3.1)	0.744
Death, n%	2 (0.6)	1 (1.0)	1 (0.4)	0.525
Myocardial infarction, n%	5 (1.5)	1 (1.0)	4 (1.7)	1.000
Emergency revascularization, n%	1 (0.3)	0 (0)	1 (0.4)	1.000
Cardiogenic shock, n%	3 (0.9)	1 (1.0)	2 (0.9)	1.000
Cardiac arrest/ventricular Fibrillation, n%	3 (0.9)	2 (1.9)	1 (0.4)	0.228
Stroke, n%	2 (0.6)	0 (0)	2 (0.9)	1.000

NSTEMI: non-ST segment elevation myocardial infarction; PCI: percutaneous coronary intervention.

Supplementary Table 4. The rates of MACEs in subgroups of NSTEMI patients undergoing CAG

	No. of Patients	Early CAG	Delayed CAG	*P* Value
Age, n (%)				
≥75 yr	56	0 (0.00)	2 (3.57)	1.000
<75 yr	445	4 (0.91)	8 (1.81)	0.760
Sex, n (%)				
Female	156	0 (0.00)	5 (3.21)	0.588
Male	345	4 (1.16)	5 (1.45)	0.488
Heart failure, n (%)				
No	447	2 (0.45)	7 (1.57)	0.727
Yes	54	2 (3.70)	3 (5.56)	0.266

NSTEMI: non-ST segment elevation myocardial infarction; CAG: coronary angiography; MACEs: major adverse cardiac events.

Supplementary Table 5. Bleeding Complications in NSTEMI patients undergoing CAG, Classified by Type

	Early CAG (n=150)	Delayed CAG (n=351)	*P* Value
All, n (%)	17 (11.3)	24 (6.8)	0.109
Gastrointestinal hemorrhage, n (%)	2 (1.3)	5 (1.4)	1.000
Epistaxis, n (%)	1 (0.7)	3 (0.9)	1.000
Ecchymoma, n (%)	4 (2.7)	6 (1.7)	0.496
Urological hemorrhage, n (%)	8 (5.3)	5 (1.4)	0.026
Gum bleeding, n (%)	1 (0.7)	3 (0.9)	1.000
Bloody sputum, n (%)	0 (0)	2 (0.6)	1.000
Menorrhea, n (%)	1 (0.7)	0 (0)	0.299

NSTEMI: non-ST segment elevation myocardial infarction; CAG: coronary angiography

Supplementary Table 6. Procedural complications of NSTEMI patients undergoing PCI

	Early PCI (n=103)	Delayed PCI (n=229)	*P* Value
All, n (%)	15 (14.6)	24 (10.5)	0.357
Hemodynamic or electrical instability, n (%)	3 (2.9)	7 (3.1)	0.707
Coronary artery dissection, n (%)	0 (0)	2 (0.9)	1.000
Coronary artery spasm, n (%)	0 (0)	1 (0.4)	1.000
Entrapped equipment, n (%)	1 (1.0)	0 (0)	0.301
Stent malposition, n (%)	3 (2.9)	0 (0)	0.029
Stent under-expansion, n (%)	5 (4.9)	7 (3.1)	0.526
Coronary no-reflow, n (%)	2 (1.9)	1 (0.4)	0.228
Peripheral vascular injury, n (%)	1 (1.0)	6 (2.6)	0.443

NSTEMI: non-ST segment elevation myocardial infarction; PCI: percutaneous coronary intervention
